# mTOR Signaling and Potential Therapeutic Targeting in Meningioma

**DOI:** 10.3390/ijms23041978

**Published:** 2022-02-10

**Authors:** Benjamin Pinker, Anna-Maria Barciszewska

**Affiliations:** 1 Medical Faculty, Karol Marcinkowski University of Medical Sciences, Fredry 10, 61-701 Poznan, Poland; 2 Intraoperative Imaging Unit, Chair and Department of Neurosurgery and Neurotraumatology, Karol Marcinkowski University of Medical Sciences, Przybyszewskiego 49, 60-355 Poznan, Poland; ambarciszewska@ump.edu.pl; 3 Department of Neurosurgery and Neurotraumatology, Heliodor Swiecicki Clinical Hospital, Przybyszewskiego 49, 60-355 Poznan, Poland

**Keywords:** meningioma, mTOR, redox homeostasis, macroautophagy, everolimus, vistusertib, lycopene

## Abstract

Meningiomas are the most frequent primary tumors arising in the central nervous system. They typically follow a benign course, with an excellent prognosis for grade I lesions through surgical intervention. Although radiotherapy is a good option for recurrent, progressive, or inoperable tumors, alternative treatments are very limited. mTOR is a protein complex with increasing therapeutical potential as a target in cancer. The current understanding of the mTOR pathway heavily involves it in the development of meningioma. Its activation is strongly dependent on PI3K/Akt signaling and the merlin protein. Both factors are commonly defective in meningioma cells, which indicates their likely function in tumor growth. Furthermore, regarding molecular tumorigenesis, the kinase activity of the mTORC1 complex inhibits many components of the autophagosome, such as the ULK1 or Beclin complexes. mTOR contributes to redox homeostasis, a vital component of neoplasia. Recent clinical trials have investigated novel chemotherapeutic agents for mTOR inhibition, showing promising results in resistant or recurrent meningiomas.

## 1. Introduction

Approximately 70,000 brain tumors are diagnosed each year in the United States, of which meningiomas are the most frequent, accounting for 36% of adult tumors [1]. NHS England reports, on average, 2000 new cases of meningioma, with over half of such patients requiring surgical intervention [2]. According to the WHO classification, meningiomas can be subclassed as grade I, II or III, and further subdivided into 15 histologic subtypes [3]. The majority of cases are classified as benign and exhibit a small five-year recurrence rate of 10% [4]. Nevertheless, grade II and III meningiomas are more aggressive and exhibit higher recurrence rates of 50% and 80%, respectively [5]. Such cases may require secondary surgeries which are associated with significant complication rates. Furthermore, tumor location contributes to the difficulty of complete resection. Skull-based lesions or tumors that infiltrate the parasagittal sinuses are frequently difficult to totally resect and necessitate effective alternative treatments [6,7]. Certain patients are unfit to undergo surgery due to advanced age or comorbidities. The main non-surgical treatment is radiotherapy, which achieves disease stabilization in most cases but rarely tumor regression. Overall, despite the high success rate of benign meningioma resection, a subset of patients desperately requires new options [7].

Treatment approaches for meningioma are unique among central nervous system neoplasms, as the arachnoid cells within the meningeal layer are located outside the blood–brain barrier which widens the possibility of chemotherapeutic drugs [8]. Nevertheless, the treatment options for therapy-resistant meningiomas are severely underdeveloped [9]. The 6-month progression-free survival rate (PFS6) is estimated to be 11–15% in untreated recurrent tumors. Due to the poor prognosis, novel treatments displaying a PFS6 over 35% are considered to be of interest [10].

The mechanistic target of rapamycin (mTOR) is a protein kinase, belonging to the PI3K-related kinase family which functions in gene regulation and in control of progression through the cell cycle from the G1 to S phase [11]. The protein kinase forms the core catalytic component of two protein complexes, mTORC1 and mTORC2. Although both complexes share this core component, they possess a unique subunit composition and differ in their subcellular localization, substrate binding, and regulation [12]. Both complexes integrate a multitude of molecular signals which contribute to the regulation of cell survival, cell growth, cell metabolism, protein synthesis, and autophagy [13]. The ensuing downstream events are important to the tumorigenesis of many cancers [14]. Currently, the FDA has approved mTOR inhibitors in the management of several cancers (Table 1) [15,16,17,18].

## 2. The mTOR Complex 1 (TORC1)

The tumorigenesis of meningioma involves several pathways with resultant downstream mTORC1 activation. Two noteworthy genes include the loss of NF2 and mutations in Akt, an important component of the PI3K/Akt pathway. Both mechanisms have been shown to activate the mTORC1 complex independently of one another [19,20].

### 2.1. The NF2 Pathway

On the other hand, the most common tumor suppressor gene effected in meningiomas is the NF2 gene, encoding Merlin. Approximately 60% of sporadic tumors exhibit NF2 loss. Merlin is a member of the ezrin-radixin-moesin (ERM) family which functions as a link membrane protein to the intracellular actin cytoskeleton. The exact biological mechanism of merlin is incompletely understood [21]. The protein is located in the cortical cytoskeleton region of the cell and switches between closed and open formation [22]. The activity of the molecule is controlled by a series of pathways that integrate various growth factors [23]. Furthermore, contact inhibition is an additional mechanism that enacts molecular modulation of NF2 to prevent or promote cell growth [24,25,26,27,28]. A wide variety of downstream pathways are involved in the control of proliferation, and its relation to mTORC1 [19,21].

**Figure 1:**
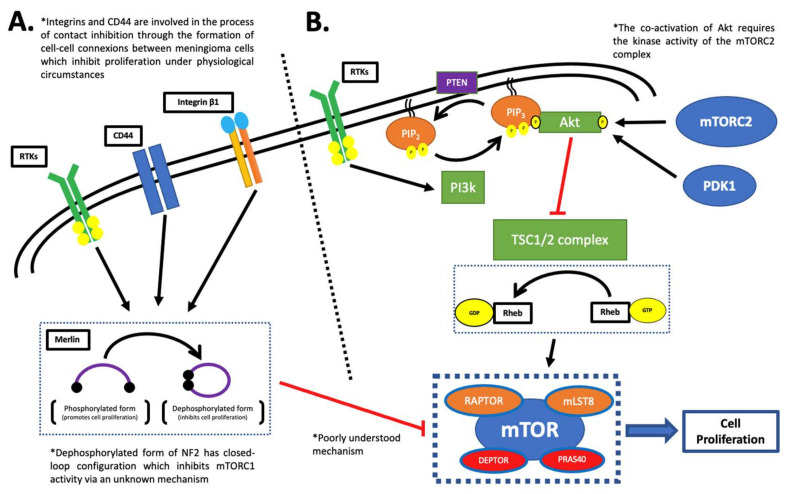
The activation of the mTORC1 complex. (**A**) The loss of NF2 is associated with increased mTORC1 activity via an unknown mechanism [22,23]. Contact inhibition, an important mechanism in meningeal cells, is reported to be involved in the upstream regulation of the protein. The contact inhibition is mediated by multiple proteins, such as RTKs, CD44, and integrin beta-1 [24,25,26,27,28]. (*) The ensuing activation of the merlin protein leads to mTORC1 inhibition through a poorly understood mechanism [22,23]. (**B**) The PI3K/Akt/mTOR pathway is a critical pathway in cell proliferation. The pathway is triggered by growth factors stimulative of RTK receptors [32]. The ensuing steps result in control of the TSC1/2 complex, the principal regulator of mTORC1 complex activity. TSC1/2 acts via Rheb, which activates the mTORC1 complex in the GTP-bound form to drive cell proliferation [31,32,33,34,35,36].

The relevance of NF2 lies in the fact that there is a firm correlation between merlin deficiency and heightened mTOR pathway activity. However, the exact mechanism linking the mTOR pathway to NF2 is poorly understood (Figure 1A) [19]. Multiple studies have suggested different possible mechanisms such as direct activation of TSC1/2 complex. Supporting evidence indicates that TSC1 interacts with member of the ERM protein family and both molecules share overlapping enzymatic activity [19]. Alternatively, it is proposed that merlin inhibited mTOR activity through a ubiquitin ligase enzyme, CTLA4. The data suggested that merlin directly inhibited proliferation by recruiting two substrates, DCAF1 and VprBP to CTLA4. Moreover, the mTOR inhibitor, TSC2, is known to be targeted by CTL4 for ubiquitination and subsequent proteasomal degradation. These molecular models reveal potential insight into the downstream signals of NF2 [29].

However, this model challenged with observations which showed that CRL4 inhibitors had no effect on the mTOR activity levels [30]. Overall, the tumorigenesis in meningioma remains poorly understood and warrants further research.

### 2.2. The PI3K/Akt Pathway

The PI3K/Akt pathway is a signal transduction cascade heavily involved in cell proliferation. It is linked with growth factor stimulation via receptor tyrosine kinase (RTK) receptors such as insulin or epidermal growth factor receptor [31]. Class I PI3K enzymes are the main downstream effectors of RTKs and function by converting phosphatidylinositol (4,5)-bisphosphate to phosphatidylinositol (3,4,5)-trisphosphate through their kinase activity. The protein kinase B protein, Akt, requires dual phosphorylation for activation. PIP3 localizes Akt to the plasma membrane and phosphorylates threonine 308 and serine 473 residues through mTORC2 [32]. This complex is speculated to act as a secondary regulator of mTORC1 though an incompletely understood mechanism which bypasses the PI3K/Akt pathway. This is further discussed in the mTORC2 complex section [33]. The subsequent activation of Akt induces the downstream effector, TSC1/2 complex. This is the principal regulator of mTORC1 which functions through the Rheb protein by hydrolysis of GTP to GDP. This molecule exists in an active and inactive complex with GTP and GDP, respectively. The pathway is illustrated in detail in Figure 1B [34].

It has been found that PI3K/Akt pathway is widely accepted as an upstream regulator of mTORC1 complex activity. Moreover, the TSC1/2 complexes are downstream component of this pathway which acts as the principal regulatory step through the Rheb GTPase enzyme [35,36]. It has been shown that the PI3K/Akt pathway and mTOR are heavily linked to the redox homeostasis of neoplastic cells, a critical component in the development of tumors [37].

## 3. The mTOR Complex 2 (mTORC2)

mTORC2 is insensitive to rapamycin inhibition, which was the first discovered mTOR inhibitor. Furthermore, the activation pathways and downstream activity of the mTORC2 complex are less well established due to the absence of specific inhibitors. Nevertheless, it is well established to be involved in yeast cytoskeleton organization [38].

The upstream regulation of mTORC2 activity is the least well understood aspect in comparison with the mTORC1 complex. The strongest evidence links it with the PI3K/Akt/mTOR pathway through PIP3, which has been shown to activate the mTORC2 complex via an indirect pathway. This relationship is not conclusive, although this pathway also results in downstream regulation of the mTORC1 complex (Figure 1) [39]. The Akt protein is the best characterized target of the mTORC2 complex. The activity of Akt depends on the phosphorylation status at two different residue sites, threonine and serine. One of them is the serine 473 residue. However, conflicting evidence exists surrounding the nature of the interaction between mTORC2 and Akt and the relation with growth factor stimulation [39,40].

The cellular localization of the mTORC2 complex within the cell appears to be an additional factor that determines the level of activity. The phosphorylation state at the Ser473 in Akt has been used to quantify the mTORC2 activity levels [41]. Overall, mTORC2 activity was concentrated at the plasma membrane, mitochondria, and a subpopulation of endosomal vesicles. The phosphorylation of Akt is elevated independently of growth factor-induced PI3K activation at the plasma membrane compared to the other sites. This implies that mTORC2 may be constitutively active and drive phosphorylation of Akt independently of growth factor stimulation. The different circumstances of mTORC2 activation around the cell may account for the conflicting evidence [41].

The mTOR complexes exhibit relatively independent functions, nevertheless, a feedback loop integrates their activity. The mTORC1 complex acts as a negative feedback regulator via downstream components, S6K1 and Grb10. These factors lead to the inhibitory phosphorylation of IRS1, which results in the inhibition of mTORC2. Furthermore, S6K1 has been shown to directly phosphorylate RICTOR and mSin1 of the mTORC2 complex. This provides insight into the interplay between mTORC1 and mTORC2. Selective mTORC1 inhibitors increase Akt phosphorylation at Ser473, reflecting elevated mTORC2 activity, and therefore explaining the superior results associated with non-selective mTOR inhibitors [42].

There is evidence for the specific role of mTORC2 in meningioma cells. However, the NF2 protein positively regulates the phosphorylation of mTORC2 substrates, including Akt. NF2-deficiency is the most prominent mutation in meningioma. Growth factor stimulation of NF2-deficient cells produces a weaker Akt activation response. These findings were only present in normal arachnoid and Schwann cell lines. Subsequently, it appears that meningioma and schwannomas tumors acquire additional molecular defects that prevent mTORC2 attenuation [42].

## 4. mTOR Inhibitors

First-generation mTOR inhibitors are derivatives of rapamycin (also known as rapalogs). They principally target mTORC1, with no activity against mTORC2. However, prolonged exposure to rapamycin has been shown to inhibit mTORC2 by preventing complex assembly [43].

**Figure 2:**
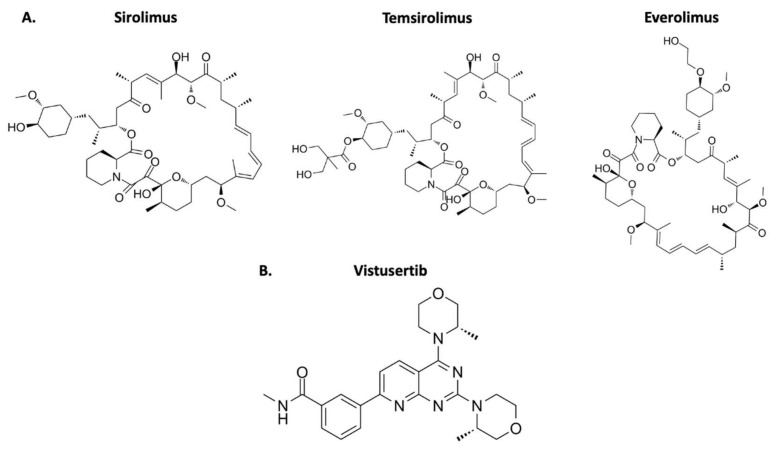
Chemical structure of mTOR inhibitors approved by the FDA. (**A**) The first-generation mTOR inhibitors that target mTORC1. (**B**) The second-generation mTOR inhibitor that targets mTORC1 and mTORC2. It is not yet FDA-approved for any treatment course [47,48,49,50].

Everolimus, a first generation mTOR inhibitor, is the more frequently used option in cancer treatment (Table 1) [14]. A handful of clinical trials have used this therapy to investigate meningioma treatment. It is important to note that this rapalog shows the strongest activity against mTORC2 amongst the first-generation drugs (Figure 2A). The inhibition is minimal but may contribute to reducing feedback activation of the complex [43]. The second-generation mTOR inhibitors have strong activity against mTORC1 and mTORC2 complexes and may have potential as an effective treatment (Figure 2B). Currently, a clinical trial is investigating vistusertib in refractory meningioma (ClinicalTrials.gov/NCT03071874).

The argument for using mTOR inhibitors in the management of meningioma must be counterbalanced with the potential adverse effects. Among the different mTOR inhibitors available, everolimus has been the most widely used in oncology and was utilized in the published in vivo work on meningioma thus far. A series of BOLERO trials studied the effectiveness of everolimus in breast cancer and elaborated on the toxicity profile [44]. A review of all the data established a similar toxicity profile in the different trials. The most frequent adverse effects included stomatitis, diarrhea, rash, fatigue, nausea, decreased appetite, weight loss, cough, dyspnea, and anemia. These symptoms were usually classified as mild to moderate and rarely led to interventions, such as dose modifications or treatment disruption. Although rare, certain patients developed noninfectious pneumonitis, which is a life-threatening condition [45].

The discontinuation of everolimus administration in patients with solid tumors recorded a relative risk of 2.60. However, it is difficult to interpret this value as it varied significantly among tumor types. Additional factors, such as dosage or therapy combinations, did not correlate with everolimus discontinuation. The most frequent events were fatigue, pneumonitis, stomatitis, diarrhea, neuropathy, and hemorrhage [45]. Overall, studies on the toxicity of everolimus have found this chemotherapeutic agent to be well tolerated [46].

Overall, the therapeutic targeting of mTOR in chemotherapy relies principally on the inhibition of mTORC1. Nevertheless, an understanding of the biology underlying mTORC2 can explain drug resistance and could lead to the development of effective alternatives [51].

## 5. Redox Homeostasis and mTOR

Maintenance of the cell at the redox state is the most important issue for cell homeostasis. The PI3K/Akt/mTOR pathway is unique since it promotes both the generation and detoxification of oxidative stress in order to drive tumorigenesis without destabilizing the cell. It holds a central role in tumorigenesis and controls many of the hallmarks of cancer. The balance between both states lies in the fact that reactive oxygen species (ROS) promote tumor growth through cell damage, such as DNA mutations, post-translational changes to proteins, and plasma membrane defects. On the other hand, excessive oxidative damage destabilizes the cancerous cells and leads to eventual death. Thus, cancerous cells must strike a balance between both states [37].

Most oxidative stress arises during energy production in the mitochondria. These organelles perform oxidative phosphorylation to create ATP molecules and ROS as by-products. Therefore, the turnover of mitochondrial organelles is crucial for redox homeostasis since mitochondrial dysfunction is intrinsically linked with elevated oxidative stress. Furthermore, brain cells are highly abundant in mitochondria in order to meet the energy demands, which leaves them very vulnerable to this oxidative damage [52].

### 5.1. mTOR Control

**Figure 3:**
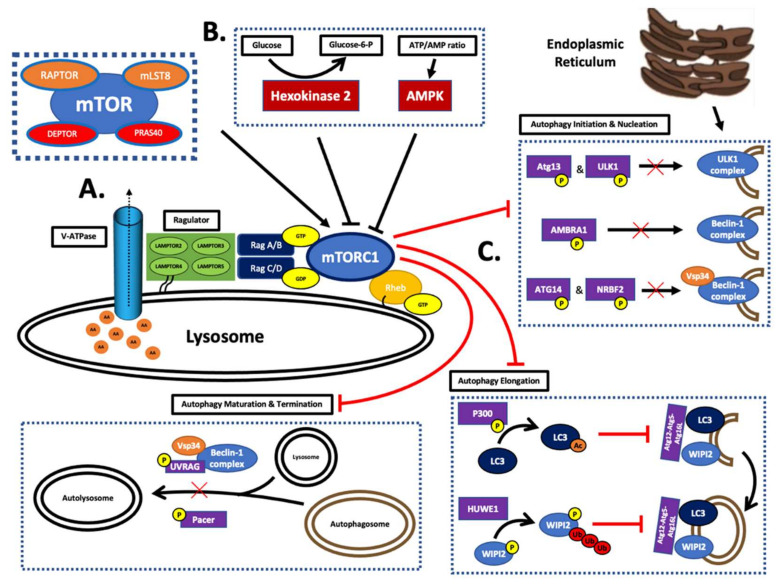
The role of mTORC1 in the control of autophagy. (**A**) The activation process of mTORC1 occurs at the lysosomal surface. It involves the detection of protein degradation in the lysosome by Rag, which is bound to the lysosome by Ragulator. The amino acids are released into the cytosol by V-ATPase. The activation of mTORC1 further requires the recruitment of Rheb in the GTP-bound form [31,53]. (**B**) The complex regulation of mTORC1 includes the incorporation of the nutritional status of the cell. Under low glucose conditions, enzymes in the glycolysis pathway inhibit mTORC1. Hexokinase can directly phosphorylate the mTORC1 complex and drive autophagosome formation to produce energy for the cell through lysosomal degradation. Furthermore, AMPK has also been shown to inhibit the mTORC1 complex. It reflects the ATP:AMP ratio of the cell [31]. (**C**) The mTORC1 complex prevents the formation of the autophagosome by the phosphorylation of multiple factors required for activation. During initiation, the pre-autophagosome buds off the endoplasmic reticulum. This structure is bound by the ULK1 complex that is constitutively assembled unless inhibited. The formation of this multi-protein molecule is regulated by mTOR activity. The mTORC1 complex targets two different subunits for inhibition, ATG13 and ULK1. Phosphorylation of either subunit prevents ULK1 complex formation and the subsequent phagophore. The following phase is known as nucleation and is conducted by Belcin1. This secondary protein complex is activated via ULK1 complex-induced phosphorylation. As a result, this complex begins generating PIP3 on the surface of the phagophore through its class III PI3K enzyme activity. The kinase function is performed by the catalytic subunit, Vps34. Under nutrient-rich conditions, mTORC1 inactivates the Belcin1 complex through the phosphorylation of Atg14, AMBRA1, or NRBF2. Autophagosome expansion is directed by the class III PI3K; it enables WIPI2 attachment to the surface of the phagophore. Subsequently, WIPI2 recruits the large protein complex Atg12–Atg5–Atg16L to facilitate LC3 binding via the Atg16L subunit. It is important to note that LC3 is crucial for assembling and completing the double membrane of the phagolysosome. The mTORC1 complex enacts its control on the elongation phase through WIPI2 and p300. The inhibition of LC3 is unique as it occurs via p300-catalysed acetylation, which prevents it from exiting the nucleus. Furthermore, WIPI2 is directly phosphorylated by mTOR, which promotes ubiquitin ligase targeting and eventual breakdown [31].

The PI3K/Akt/mTOR pathway is very important in regulating macroautophagy. This type of autophagy is involved in the degradation of large structures, such as mitochondria, through the formation of autophagosomes [53]. The activation of mTOR and its relationship with autophagy is largely dependent on the detection of nutrients, growth factors, and glucose through an elaborate system (Figure 3B). The main activation site of mTORC1 is localized at the surface of the lysosome (Figure 3A). The GTPase heterodimers, RagA-D, drive the translocation and binding of mTORC1 to the surface of the organelle. In addition, Ragulator is important for binding the Rag proteins to the lysosome and functions in the identification of amino acids (Figure 3A) [31]. The channel protein V-ATPase releases amino acids into the cytosol from degraded proteins within the lysosome [53]. A secondary signal from Rheb is required for mTORC1 complex activation (Figure 3A). This enzyme detects amino acids and phosphorylates mTORC1 through its kinase activity depending on this nutritional state. The ensuing phagosome inhibition is achieved through the inactivation of a multitude of enzymes (Figure 3C) [31].

The energy status of the cell is controlled by the regulation of mTORC1 via hexokinase 2 and AMPK. The glycolytic enzyme hexokinase 2 phosphorylates glucose to glucose-6-phosphate. In an energy-deficient state, low glucose stimulates hexokinase to bind and deactivate the mTORC1 complex. Furthermore, mTOR activity is monitored by AMPK depending on the ATP:AMP ratio within the cytosol. Under conditions of low ATP, AMPK inhibits the mTORC1 complex to drive phagolysosome formation [31].

### 5.2. Macroautophagy

Macroautophagy is a multi-step process with the formation of a double-membrane organelle known as an autophagosome. The endoplasmic reticulum provides a plasma membrane for engulfing targeted cytoplasmic components. Subsequently, the autophagosome fuses with a lysosome to form an autolysosome for the destruction of engulfed material (Figure 3C) [31].

The initiation of macroautophagy begins with the budding of a pre-autophagosome from the endoplasmic reticulum. The process may be divided into five separate phases consisting of initiation and nucleation, elongation, maturation, and termination. Throughout this complex system, mTORC1 directly controls many proteins involved in autophagosome formation through its kinase activity. The phosphorylation of many factors, such as ULK1, Atg13, P300, and HUWE1, results in pathway inhibition (Figure 3C) [31].

In the final phase of autophagosome assembly, the mTOR1 complex phosphorylates UVRAG and Pacer, which inhibit fusion with a lysosome to inhibit the later stages of phagolysosome assembly. As with previous phases, mTOR-induced phosphorylation depends on the adequate nutritional status of the cell. Overall, the mTORC1 complex regulates eight different proteins during macroautophagy assembly through phosphorylation and one additional enzyme through acetylation, LC3 (Figure 3C) [31].

### 5.3. The Thioredoxin–Thioredoxin Reductase System

Thioredoxin–thioredoxin reductase (TRX-TRXR) is an additional pathway that plays a major role in redox homeostasis. The enzymatic reaction produces the reducing factor, NADPH. Thus, the system has been found to be upregulated in meningioma cells. Inhibition of system components have been shown to abolish tumor growth in other cancers [54]. Chaetocin is a small molecule thiodioxopiperazine that appears to inhibit the system. The treatment induced enriched PI3K/Akt pathway activity, which resulted in mTOR activation [55]. It would be interesting to investigate the potential for chaetocin treatment in meningioma. Furthermore, a deeper understanding of its interaction with the PI3K/Akt pathway would be valuable.

Overall, the mTORC1 complex appears to be a critical inhibitor of the formation of autophagosomes through the regulation at many stages of the activation pathway. This function ties into the role of mTOR with the redox homeostasis of meningioma cells. It is a major protective mechanism against oxidative stress which will maintain tumor stability and growth. Thus, targeting mTOR activity in meningioma has the potential to disrupt many crucial parts of tumor viability [37,55].

## 6. Therapeutical Application

The management guidelines established by evidence-based medicine for meningioma are much less developed in comparison to other central nervous system tumors. The European Association of Neuro-Oncology (EANO) published recommendations on the management of meningioma. Surgical resection is the first-line treatment in the majority of meningiomas. Nevertheless, the approaches are individualized based on symptoms, size, location, growth rate, calcifications, hyposignal intensity on imaging, and the potential consequences of surgery [56].

The WHO 2021 grading for meningiomas classifies tumors into three grades based on histology, biomarkers, and clinical features. More emphasis was placed on biomarkers as opposed to morphological features in the 2021 guidelines [3,57]. The grade I tumors are managed depending on many factors. An observational approach is a viable option for grade I meningiomas only. Patients are monitored for symptoms and tumor growth for six months followed by yearly MRI imaging check-ups. Surgical intervention is the only therapy with a curable potential. Patients presenting with mass-induced symptoms or radiological evidence of growth are recommended to undergo surgery as a first-line treatment. However, small tumors may undergo stereotactic radiosurgery in certain cases, such as patients over 65 years of age, inaccessible tumors, or following incomplete surgical resection. In the latter scenario, a combinational approach has been increasingly utilized to protect sensitive neurovascular structures, such as the cavernous sinus. Studies have shown that the follow-up assessments report lower levels of neurological deficit when combining partial surgical resection with stereotactic radiosurgery. Alternatively, larger tumors will require fractionated radiotherapy to supplement the subtotal resection [56].

The management approaches for WHO grades II and III meningiomas are even less definitive. The rates of recurrence and metastasis are significantly higher, warranting more aggressive approaches. The first-line treatment consists of maximal surgical resection with adjuvant radiotherapy in fractionated doses for all patients. However, the data is largely inconclusive beyond total tumor removal. Although adjuvant radiotherapy is recommended, many trials have tested different radiotherapy regimens without establishing concrete guidelines. In terms of chemotherapy, many small retrospective studies have examined a multitude of drugs with various mechanisms of action. The outcomes and effectiveness of chemotherapy regimens vary widely. Overall, pharmacotherapy is recommended for patients with progression despite surgery or radiotherapy, inoperable cases, and elderly patients [56].

Herein lies the clinical potential for drugs targeting the mTOR pathway. The mTOR complex is a component of the PI3K/Akt pathway, which was first established as a component of the meningioma tumorigenesis mechanism through western blot studies by Johnson et al. [58,59]. Furthermore, NF2 is a commonly defective gene in meningioma and is understood to be a negative regulator of mTOR, therefore NF2 deletion will drive meningioma proliferation [19,33,60]. As a consequence, in vitro data suggest that mTOR inhibitors may be beneficial in treating meningioma.

Very few in vivo studies have been conducted on mTOR inhibitors in meningioma treatment. Previous research on preclinical models from other cancers have elucidated a role for mTOR inhibitors in cancers, such as myeloma or prostate cancer [61,62]. The first relevant publication involved the injection of human WHO stage III meningioma cells into mouse models. The cell lines were injected into the subarachnoid space to replicate physiological conditions. Subsequently, the mice received systemic chemotherapeutic treatment with two mTOR inhibitors, sirolimus and temsirolimus. The study found that both inhibitors were clearly effective in reducing meningioma cell viability and proliferation. In addition, western blot analysis showed that the response was due to the specific inhibition of mTORC1 activity (Table 2A) [21].

A handful of drug trials and a case study have been published over the past five to ten years. Bertolini et al. presented a case report of a women who was diagnosed with a WHO grade I meningioma in the right frontoparietal sagittal sinus. The tumor had metastasized to the lungs and multiple bilateral cystic pulmonary lesions were seen on the chest CT. The patient underwent treatment with hydroxyurea, which was ineffective as it resulted in an increase in number and size of the cystic pulmonary lesions. Genetic analysis was positive for phospho-mTOR and phospho-p70S6 and therefore, in this exceptional case, the patient underwent everolimus therapy with ensuing positive results directly related to the treatment. In particular, the chest CT showed stable disease and no relapse. In addition, no relapse of the primary brain tumor was reported with MRI (duration of response: 27 months) (Table 2) [63].

The first human trial to include an mTOR inhibitor was an investigation into a combination therapy of everolimus and bevacizumab (VEGF-A inhibitor). This phase II prospective trial included a cohort of 18 symptomatic grade I, II, and III progressive or refractory meningiomas. The patients had to meet certain requirements, such as a history of previous surgical resection or radiotherapy. The results found that 88% of patients produced stable disease (15/17); moreover, the duration lasted over 12 months in six of the 15 patients. Overall, the combination therapy of everolimus and bevacizumab produced prolonged disease stability; however, no tumor response was reported (Table 2 (C)) [64].

The results of a phase II clinical trial involving 20 patients were very promising. This was a prospective research trial which included meningiomas ineligible for further surgery or radiotherapy. In addition, the tumors were required to have a documented progression over a 3-month or 6-month period. The treatment was a combination therapy regimen of everolimus (10 mg/day) and octreotide (30 mg/month) [10]. Octreotide is a somatostatin analogue that is used in the treatment of growth factor-dependent tumors. It is important to note that a previous report showed that octreotide therapy in meningioma is inversely correlated with the activation of mTOR downstream effectors (S6K) [65]. The phase II trial demonstrated a decrease (>50%) in the volume growth rate in 78% of patients at 3 months and in 67% at 6 months. Moreover, among the 20 patients studied, a decrease (>10%) in the tumor volume was observed in four patients at 3 and 6 months. The results in the trial strongly suggested that everolimus and octreotide therapy is effective in treating aggressive meningiomas (Table 2 (D)) [10].

A study correlated gene expression with meningioma tumor location within the skull. Skull-based tumors in the base of the cranium are particularly difficult to surgically resect. Therefore, a comprehensive understanding of gene expression may help develop targeted therapies. The data showed that Akt mutations were present at a higher frequency. In addition, the mutation was associated with a shorter delay of tumor recurrence [66]. The Akt gene is a major regulatory component of the PI3K/Akt pathway which results in downstream activation of the mTORC1 complex [59]. Thus, mTOR inhibitors may have the potential to benefit patients with incomplete resection of skull-based meningiomas [66].

According to ClinicalTrials.gov, there are two ongoing clinical trials examining the effects of mTOR inhibitors in meningioma patients. In particular, a single arm phase II trial by led by S. Plotkin at Massachusetts General Hospital is evaluating vistusertib in grade II–III meningiomas that have recurred or progressed after surgery and radiation (ClinicalTrials.gov/NCT03071874). Dual mTOR complex inhibition is in line with the in vitro understanding of the interplay and feedback mechanisms between mTORC1 and mTORC2 (Table 3 (B)).

## 7. Application as a Biomarker

Prognostic factors in brain tumors are greatly sought after. Currently, meningioma prognosis principally relies on histological grade and surgical resection. However, alternative prognostic factors are lacking in cases of radical surgical resection or treatment-resistant cases. Such factors may be greatly beneficial in atypical meningiomas, which exhibit a highly varied clinical behavior. This is illustrated by the tumor’s oncological grading, which ranges from WHO grade I to III [67].

Barresi et al. correlated mTOR activation with serine phosphorylation in position 2448 (p-mTOR Ser2448) [9]. Among the cohort, high p-mTOR expression was significantly associated with recurrence in atypical meningioma. In addition, disease-free survival was found to be lower with high p-mTOR expression [67].

Nevertheless, previous studies have been conducted on the potential of mTOR as a prognostic factor with limited results, though this study provided no comparison with different meningioma histotypes and did not provide any quantitative data on mTOR expression [68,69].

## 8. Prospective

The review provided much evidence linking the mTOR pathway to the pathogenesis of meningioma. Exploring the published data concerning drugs that target the mTOR complex, in vitro and in vivo studies have both produced promising results. Targeting the pathway through natural products with minimal side-effects may be equally effective.

### 8.1. Lycopene Treatment

The carotenoid lycopene, the most potent antioxidant, is a natural product widely found in tomatoes, grapefruits, pomegranate, and watermelons [70]. As previously described, mTOR is heavily involved in the redox homeostasis of the cell. Given mTOR’s function in controlling the level of cellular reactive oxygen species, an antioxidant such as lycopene may disrupt this homeostasis. Furthermore, lycopene is understood to have anti-proliferative activity in neoplastic disorders, such as prostate cancer and oral cancer. Recent studies have suggested that mTOR inhibition may be a contributing factor [71,72,73]. An in vitro study on a human cell line of oral cancer cells concluded that lycopene suppressed the PI3K/Akt/mTOR pathway in a dose-dependent manner. Additionally, the data suggested that it promoted apoptosis in this cell line [73]. It is important to note that the evidence is not overwhelming, as other studies report increased PI3K/Akt/mTOR pathway activation with lycopene treatment, although this study was conducted on varicocele rats and not in cases of oncological disease [74]. Further studies conducted in vivo on laboratory mice established a direct link between mTORC1 complex inactivation and lycopene administration. Direct mTORC1 RNA was measured after lycopene dietary supplementation for 24 weeks in hepatocellular carcinoma development in obesity [75]. Lastly, lycopene may be advantageous over first generation mTOR inhibitors, as it non-selectively suppresses both complex subtypes. Although second generation mTOR inhibitors are available, they are very toxic [76].

Lycopene supplementation has been most widely assessed in the clinical context of prostate cancer. Approximately 25 clinical trials are currently documented on ClinicalTrials.gov, with additional trials being conducted on breast and endometrial cancer. The PI3K/Akt/mTOR pathway is well known to be hyperactive in prostate cancer. Furthermore, elevated levels of mTOR expression are understood to contribute to tumorigenesis. mTOR expression was shown to be higher in cancerous prostate cells [77,78,79]. Subsequently, the potential role for mTOR inhibitors has been investigated through in vitro studies and clinical trials. However, the results in the clinical trials have produced a limited response. This is likely due to complex feedback systems and limited activity of mTORC2. Further studies have combined mTOR inhibitors with suppressants of other members of the PI3K/Akt/mTOR pathway with pending results [78].

Lycopene therapy has mostly been studied in prostate cancer, a malignancy shown to involve mTOR signaling [77,78,79]. Most publications have focused on dietary supplementation as a protective or therapeutic measure [80,81]. Overall, a series of studies on lycopene in prostate cancer have demonstrated positive results [82,83,84,85,86,87].

### 8.2. Methylation Targeting

Many components of the downstream activity of the mTORC1 complex are yet to be fully understood. Recent studies suggest that mTOR plays a role in controlling the epigenetic state of the cell [88]. Epigenetics refers to the control of gene expression without modifying DNA sequences and is heavily implicated in all cancers [86]. The interplay with mTORC1 is suggested by its control of 1C metabolism, which produces SAM (S-adenosyl methionine), a critical substrate for DNA and histone methylation. In particular, mTORC1 leads to H3K27 hypermethylation in glioblastoma cells [89,90].

As discussed previously, bypassing pathways and feedback mechanisms leading to resistance are a major concern of mTOR inhibitors in cancer. Therefore, countering this through dual therapies which target mTOR substrates may have an addictive effect. Azacitidine and decitabine are two new medications that function by inhibiting DNA methyltransferase. These two cytosine derivatives are currently being investigated as chemotherapeutic regimens in hematological cancers. In addition, an in vitro study evaluated the impact of decitabine on high-grade meningioma cells. The data described a dose-dependent significant decrease in proliferation and viability. The findings were only producible in malignant, not benign, cell lines [91]. Given mTOR’s function in the control of DNA methylation, combining such drugs with mTOR inhibitors may have the potential to produce a synergistic response in meningioma.

## 9. Conclusions

Overall, this review presents the current understanding of mTOR activity in meningioma cells. In particular, it highlights the relationship between mTOR and merlin and elucidates the potential role of autophagy and redox reactions in the development of meningioma tumors. Further understanding of these mechanisms will help in the development of targeted therapies for meningioma.

Moreover, this review has shown the great potential of mTOR inhibitors in the treatment of refractory meningioma. Although the currently available data is limited, the existing clinical trials have shown very promising results. Alternative chemotherapeutic drugs have produced disappointing outcomes in this underdeveloped area. In conclusion, new clinical trials with larger cohorts are necessary to fully reveal the role of mTOR inhibitors in meningioma.

Targeting the mTOR pathway in meningioma via alternative therapies has yet to be explored. The current understanding of the molecular mechanism underlying the development of meningioma suggests that natural antioxidants, such as lycopene, may be equally effective. Exploiting the epigenetic mechanisms, like DNA methylation pathways with azacitidine and decitabine, could target the mTOR pathway at different points to further inhibit proliferation.

## Figures and Tables

**Table 1:** FDA approved mTOR inhibitors. The FDA has approved the use of mTOR inhibitors in the management of six cancers as of November 2021 [15,16,17,18].

Drug	Disease	FDA Approval
Sirolimus	Lymphangioleiomyomatosis (LAM)	May 2015
Temsirolimus	Renal cell carcinoma (advanced disease)	May 2007
Everolimus	Renal cell carcinoma (advanced disease)	March 2009
	Breast cancer (advanced HR+ tumor)	July 2012
	Neuroendocrine carcinoma Pancreatic tumor Gastrointestinal tumor Lung tumor	February 2016
	Tuberous sclerosis-associated cancers Subependymal giant cell astrocytoma (SEGA) Renal angiomyolipoma Lymphangioleiomyomatosis (LAM)	April 2012

**Table 2:** Publications on mTOR inhibitors in meningioma (complete). Comprehensive summary of all complete in vitro studies involving mTOR inhibitors in meningiomas [10,21,63,64].

Study Title	Drug	Publication Date	NTC Number
mTORC1 Inhibitors Suppress Meningioma Growth in Mouse Models (A.)	Temsirolimus	March 2013	N/A
Everolimus Effectively Blocks Pulmonary Metastases from Meningioma (B.)	Everolimus	September 2015	N/A
A Phase II Trial of Bevacizumab and Everolimus as Treatment for Patients with Refractory, Progressive Intracranial Meningioma (C.)	Everolimus Bevacizumab	June 2016	NCT00972335
Everolimus and Octreotide for Patients with Recurrent Meningioma: Results from the Phase II CEVOREM Trial (D.)	Everolimus Octreotide	February 2020	NCT02333565

**Table 3:** Clinical trials on mTOR inhibitors in meningioma. Comprehensive summary of all clinical trials currently examining mTOR inhibitors in meningioma.

Study Title	Drug	Phase	Start Date	Completion Date	Trial Identifier
AZD2014 In NF2 Patients with Progressive or Symptomatic Meningiomas (A.)	Vistusertib	Phase 2	13 July 2016	22 December 2020	NCT02831257
Vistusertib (AZD2014) For Recurrent Grade II-III Meningiomas (B.)	Vistusertib	Phase 2	17 October 2017	25 July 2024	NCT03071874

## Data Availability

Not applicable.
